# Beyond Intentionality: A Latent Class Analysis of Barriers to Prenatal Care in an Explanatory Mixed Methods Study

**DOI:** 10.3390/healthcare13131546

**Published:** 2025-06-28

**Authors:** John Kwame Duah

**Affiliations:** Department of Political Science, Health Services Administration Program, Auburn University, Auburn, AL 36849, USA; jkd0046@auburn.edu; Tel.: +1-334-844-6234

**Keywords:** prenatal care access, latent class analysis, pregnancy intentionality, explanatory mixed-method design, maternal health disparities, healthcare barriers

## Abstract

**Objective:** Utilizing the Health Care Access Barriers (HCAB) Theoretical Framework, this study examined latent profiles of barriers to prenatal care among pregnant women in Alabama and whether these profiles mediate or moderate the relationship between pregnancy intentionality and early prenatal care initiation. **Methods**: An explanatory mixed-method design was employed, integrating quantitative analysis of Alabama Pregnancy Risk Assessment Monitoring System (PRAMS) Phase 8 data (2016–2021) with qualitative insights from expert interviews. Latent class analysis (LCA) identified subgroups based on reported barriers. Multivariable logistic regression assessed the association between pregnancy intentionality and early prenatal care initiation, controlling for covariates. A Firth-penalized multivariable logistic regression model tested interaction effects. **Results:** Planned pregnancy was associated with higher odds of early prenatal care initiation (OR = 0.78, 95% CI [0.49, 1.23], *p* = 0.286), though this association was not statistically significant. Barrier profiles did not significantly moderate or mediate the relationship. The interaction term was nonsignificant (OR = 5.19, 95% CI [0.22, 828.94], *p* = 0.309), and the mediation pathway was also not supported (indirect effect = 0.012, *p* = 0.518). Expert interviews emphasized ongoing systemic and cognitive barriers that hinder timely access. **Conclusions**: Although pregnancy intentionality was not a statistically significant predictor of early prenatal care initiation, qualitative findings highlighted persistent barriers that continue to constrain access. These results underscore the need for multilevel strategies to address informational and logistical challenges. Future research should evaluate additional pathways that influence care-seeking behaviors.

## 1. Introduction

Community resource challenges and healthcare system barriers continue to influence perinatal outcomes in Alabama [1,2], exacerbating the disproportionate burden of health inequities in the state. Current literature shows that Alabama consistently ranks among the states with the highest maternal mortality and morbidity in the United States [3], with a maternal mortality rate (MMR) of 38.6 deaths per 100,000 live births in 2020 [3]. Moreover, persistent racial and geographic disparities limit timely access to obstetric care for women of reproductive age in remote and resource-scarce settings [4,5], heightening the urgency of challenges faced by rural communities and racial minorities in seeking, accessing, and receiving care during the perinatal period.

Importantly, inadequate access to prenatal care can undermine efforts to address gaps in the provision of risk-appropriate care for pregnant women [6], especially those considered high-risk pregnancies. In Alabama, this challenge is evident, as 18.1% of women receive prenatal care only from the fifth month onward or attend fewer than half of the recommended visits for their infant’s gestational age [7]. Additionally, the financial burden of uninsured women of reproductive age seeking preconception, prenatal, and postpartum care remains a critical challenge, as many must pay out-of-pocket for these essential services. For families with limited financial resources, these costs can be especially access-limiting, exacerbating disparities in maternal health access. Notably, one in six women aged 18–44 in Alabama lacks health coverage, facing significant barriers to maintaining overall well-being [8].

Pregnancy intentionality plays a vital role in shaping maternal health behaviors, especially during the timing of prenatal care initiation. The prevailing perspective is that women with planned pregnancies are more likely to seek early prenatal care, ensuring timely access to screenings, risk assessments, referrals for specialty care, and other essential interventions that improve maternal and infant health outcomes [9]. In contrast, unintended pregnancies are often associated with delayed or inadequate prenatal care utilization, contributing to disparities in perinatal health [10]. Interestingly, a systematic review and meta-analysis found that women with unintended pregnancies had 1.42 times higher odds of delayed antenatal care use and 1.64 times higher odds of inadequate care compared to those with planned pregnancies [10].

Beyond individual decision-making, systemic barriers, such as fragmented care coordination, provider shortages, and insurance limitations, further complicate access to timely prenatal services, disproportionately impacting vulnerable populations [11]. In the Southern United States, maternity care deserts and limited provider availability exacerbate these challenges, leaving many women without adequate prenatal care options [12]. A maternity care desert is an umbrella term used to describe areas with little to no access to maternity healthcare services, which may include hospitals with obstetric care, birthing centers, and more. Access to prenatal and maternal care in Alabama remains a challenge, with limited availability of maternal care providers and ongoing concerns about care quality [13]. Altogether, these structural barriers contribute to disparities in maternal health outcomes, highlighting the need for continued efforts to enhance accessibility and care coordination.

Addressing these challenges requires a comprehensive approach that integrates pregnancy planning support with structural reforms to improve prenatal care accessibility and continuity of care. The significant proportion of women in Alabama who delay prenatal care until after the first trimester highlights an opportunity to address the underlying factors contributing to preventable adverse outcomes, such as preterm birth, low birthweight, and perinatal complications. More importantly, understanding the barriers that prevent timely access to prenatal care is essential for reducing disparities in perinatal health outcomes and fostering safer, thriving communities.

These challenges call for a structured framework for evaluating maternal healthcare barriers during the perinatal period. The Health Care Access Barriers (HCAB) Model offers a valuable perspective for examining affordability, availability, accessibility, and acceptability, essential determinants of prenatal care access. Considering the complex interplay between pregnancy intentionality and systemic care limitations, HCAB provides a robust foundation for analyzing latent barriers and informing strategies to improve perinatal health outcomes in Alabama. The study’s specific aims and hypotheses are presented in Section 1.4 to guide the overall analysis and interpretation.

### 1.1. Health Care Access Barriers (HCAB) Model as a Theoretical Framework

The Health Care Access Barriers (HCAB) Model offers a comprehensive framework for evaluating the factors that influence healthcare utilization. It categorizes barriers into three modifiable domains, each operating either independently or interacting to influence access to care [14]. These domains include the following:

I. Structural barriers, which involve logistical challenges such as transportation obstacles, clinic operating hours, and provider availability. These factors significantly impact access to maternal healthcare services, particularly in resource-limited settings [15].

II. Financial barriers, which encompass affordability issues, including the cost of care, insurance access, and out-of-pocket expenses. Economic constraints often dictate whether individuals can seek essential prenatal and perinatal services [16].

III. Cognitive barriers, which relate to individual-level determinants such as pregnancy awareness, cultural beliefs, health literacy, and perceptions of healthcare accessibility. These elements shape care-seeking behaviors and can contribute to disparities in maternal health [14].

Collectively, these barriers highlight the need for multifaceted interventions that enhance healthcare accessibility by addressing affordability, availability, and awareness [15,16]. In the prenatal context, structural challenges such as clinic location and scheduling can limit early visits. Financial constraints, such as a lack of insurance coverage, often deter women from seeking timely care. Cognitive factors, including unrecognized or unintended pregnancies, can further delay care initiation, with profound consequences on maternal and infant health outcomes.

Mapping individual women’s experiences onto these three domains helps healthcare providers and policymakers to tailor interventions to local contexts, ensuring judicious allocation of resources more efficiently. This targeted approach reduces implementation gaps, strengthens care accessibility, and minimizes inefficiencies. Also, aligning interventions with specific barriers in the healthcare systems can optimize resource distribution and improve maternal health outcomes for diverse populations.

### 1.2. Rationale for Mediation and Moderation

**Figure 1:**
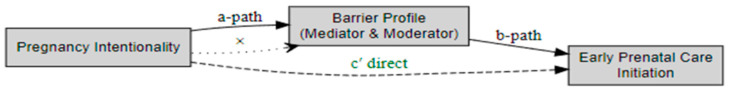
Conceptual model illustrating pregnancy intentionality, barrier profiles, and early prenatal care initiation.

To clarify this distinction, Figure 1 shows how the HCAB domains can function both as mediators and as moderators of the pregnancy intentionality → early care pathway.

Notes: Solid lines denote mediation paths (a → b), while dashed lines represent moderation effects (c′).

Although the HCAB Model was initially developed to help categorize barriers to healthcare into financial, cognitive, and structural domains, it also implies two distinct roles that those barriers can play in shaping the relationship between pregnancy intentionality and early prenatal care initiation. First, an unplanned pregnancy may increase a woman’s likelihood of experiencing access barriers (the *a-path*), which subsequently reduces the odds of timely care (the *b-path*), demonstrating a mediation process. Second, even when pregnancy is planned or intended, the extent to which it facilitates early care initiation may depend on the barrier profile, a concept known as moderation. For instance, highly motivated women may still struggle to obtain care when confronted with severe structural challenges.

This distinction is essential because mediation suggests that eliminating the barrier disrupts the causal pathway, whereas moderation highlights the need for stratified or conditional strategies tailored to different barrier intensities. In other words, mediation implies that removing the barrier disrupts the pathway from intentionality to early care, and moderation highlights when and for whom intentionality matters most, insights that guide both barrier-targeted and stratified interventions.

Recognizing these roles allows policymakers and healthcare providers to refine interventions accordingly. Addressing mediating barriers can remove obstacles to early care, while accounting for moderating effects helps create adaptive strategies suited to different levels of healthcare access limitations. Figure 1 visually represents these relationships, with solid arrows indicating mediation pathways (a → b) and dashed arrows illustrating how barrier profiles condition the direct link between pregnancy intentionality and early prenatal care initiation (c′). Previous studies have not accounted for barrier profiles as conditional determinants, requiring this approach. Expanding on the conceptual rationale for mediation and moderation, this study empirically identifies distinct barrier profiles using latent class analysis (LCA). By systematically classifying access barriers, LCA enables a nuanced understanding of how these categories influence prenatal care-seeking behaviors.

### 1.3. Latent Class Analysis of Barriers

The study now turns to the latent class analysis of barrier items. This study employs latent class analysis (LCA) on 11 barrier items from the Alabama PRAMS Phase 8 survey (2016–2021) to identify distinct subgroups of women who experience overlapping barriers to prenatal care access. In analyzing co-occurring patterns, LCA moves beyond single-variable assessment to reveal meaningful subgroup distinctions that influence pregnancy intentionality and prenatal care initiation.

Unlike traditional regression methods that examine barriers in isolation, LCA empirically identifies groups with shared access challenges, offering insights into how these profiles relate to maternal healthcare-seeking behaviors. This approach ensures a more structured assessment of how systemic and individual-level obstacles converge to shape care utilization patterns.

Applying the HCAB framework within LCA allows for the classification of women into empirically derived profiles that reflect structural, financial, and cognitive barriers. Understanding these profiles contributes to evaluating how pregnancy intentionality interacts with maternal healthcare access limitations and informs policy interventions designed to mitigate these disparities.

In grouping women into statistically derived subpopulations, LCA offers a robust framework for examining disparities in maternal health access, thereby improving interventions that address pregnancy planning support and systemic healthcare barriers in the care continuum.

### 1.4. Study Aims and Hypotheses

Building on this framework, this study seeks to answer the following research question: What latent profiles of barriers to prenatal care exist among pregnant women in Alabama, and how do these profiles influence pregnancy intentionality and early prenatal care initiation? Using data from Alabama’s PRAMS Phase 8 survey (2016–2021), this study aims to:

Identify latent barrier profiles using Alabama’s PRAMS Phase 8 barrier items.

Examine the association between pregnancy intentionality (planned vs. unplanned) and early prenatal care initiation.

Evaluate whether barrier profiles mediate and/or moderate the relationship between intentionality and early prenatal care.

Integrate qualitative insights from expert interviews to contextualize and validate quantitative findings.

The research hypotheses are as follows:

H1 (Mediation): Women with unplanned pregnancies are more likely to belong to high-barrier latent profiles, which may subsequently reduce their odds of early prenatal care initiation. If mediation is present, reducing these barriers should alter the causal pathway between pregnancy intentionality and care access.

H2 (Moderation): Even among women with planned pregnancies, early prenatal care initiation is less likely for those classified into high-barrier profiles. This suggests that pregnancy intentionality alone cannot ensure access, highlighting the need for targeted interventions to address structural and financial barriers.

H3 (Descriptive): Expert interviews will provide critical insights into how structural, financial, and cognitive barriers manifest in Alabama’s maternal healthcare system, complementing the quantitative classifications.

In situating the analysis within Alabama’s unique sociodemographic and healthcare context, and using a mixed-methods explanatory sequential design, this research aims to inform state-level policies designed to reduce maternal health disparities and improve prenatal care accessibility across diverse populations in Alabama.

## 2. Methods

### 2.1. Study Design

This study utilized an explanatory mixed-methods design, integrating quantitative analyses of data from the Alabama Department of Public Health (ADPH) PRAMS Phase 8 (2016–2021) with qualitative insights from expert interviews. The quantitative phase applied latent class analysis (LCA) to identify barrier profiles and utilized multivariable logistic regression to assess relationships between pregnancy intentionality, barrier profiles, and prenatal care initiation. The qualitative phase used thematic analysis of semi-structured interviews with field experts to provide context and inform the interpretation of the quantitative results.

### 2.2. Data Source and Sample

The quantitative data were derived from aggregated counts in the Alabama PRAMS Phase 8 dataset collected from 2016 to 2021. The original aggregate responses for key analytic variables, as detailed in Table 1, comprised 4416 unweighted observations. Due to the absence of respondent-level linkage, pseudo-individual-level data were simulated using the uncount() function in R, proportionally expanding the aggregate counts into case-level records based on unweighted frequencies.

This approach aligns with methodological standards in latent class analysis (LCA), particularly when respondent-level microdata are inaccessible due to privacy constraints or data governance policies. Existing literature supports the use of simulated data in LCA applications where aggregated structures require transformation for class estimation and model comparison. Finch and Bronk [17] showed that simulated individual-level data can be effective in evaluating latent class models across varied conditions. Similarly, Weller et al. [18] and Nylund-Gibson and Choi [19] provide guidance confirming LCA’s adaptability to diverse data formats, including constructed datasets. Muthén and Muthén [20] emphasize LCA’s flexibility in applied research settings where optimal data structures are not always feasible.

From this expanded dataset, a focused analytic sample of N = 596 was selected to reflect complete responses across key variables, which included pregnancy intentionality, prenatal care barriers, early prenatal care, and demographic covariates. Due to data-sharing restrictions, validation against individual-level PRAMS data was not feasible; however, the analytic strategy employed adheres to established guidance for exploratory analysis using transformed aggregate data. To further address concerns regarding regression modeling, Firth-penalized logistic regression was employed to mitigate bias from small cell sizes in simulated data structures. This method is widely regarded as a robust choice in contexts where data sparsity may undermine standard maximum likelihood estimation [21,22]. However, while this analytic strategy does not replace direct respondent-level microdata, it provides a reasonable and methodologically sound approximation for exploratory model testing.

The sample was selected to align with the research question, study aims, and theoretical framework (HCAB Model), ensuring analytic coherence. Weighting was not applied in this analysis due to the simulated structure of the dataset and the goal of maintaining clarity and interpretability in modeling relationships. The qualitative data were obtained through semi-structured interviews with eight experts from diverse backgrounds involved in maternal and child health policy, clinical practice, public health, social services, and healthcare administration in Alabama. These expert perspectives complement the quantitative findings by providing contextual insights into the systemic and cognitive barriers influencing prenatal care access.

### 2.3. Study Variables and Measures

A summary of all key variables, including their definitions, coding, and data sources, is provided in Table 1.

**Table 1:** Operational definitions and measurement of study variables.

Variable	Operational Definition	Coding/Measurement	Data Source
Pregnancy Intentionality	Whether the pregnancy was planned or unplanned	Planned (0), Unplanned (1)	ADPH PRAMS Phase 8; 2016–2021; Qn# 12
Early Prenatal Care	Whether care was received as early as desired	Yes (1), No (0)	ADPH PRAMS Phase 8; 2016–2021; Qn# 20
Structural Barriers	Barriers such as transportation, appointment delays, or clinic logistics	Latent class profile from LCA	ADPH PRAMS Phase 8; 2016–2021; Qn# 21
Financial Barriers	Barriers related to insurance, cost, and affordability	Latent class profile from LCA	ADPH PRAMS Phase 8; 2016–2021; Qn# 21
Cognitive Barriers	Barriers involving awareness, beliefs, and knowledge about prenatal care	Latent class profile from LCA	ADPH PRAMS Phase 8; 2016–2021; Qn# 21
Maternal Age	Age of respondent at time of birth	Categorical: 15–19 to 40+	ADPH PRAMS Phase 8; 2016–2021; Respondent Characteristics
BMI	Body Mass Index category based on pre-pregnancy weight	Underweight (n = 153), Healthy (n = 1710), Overweight (n = 1050), Obese (n = 1325)	ADPH PRAMS Phase 8; 2016–2021; Respondent Characteristics
Race/Ethnicity	Self-identified racial or ethnic group	Hispanic (1), Non-Hispanic Black (2), Non-Hispanic White (3)	ADPH PRAMS Phase 8; 2016–2021; Respondent Characteristics
Insurance Status	Type of insurance coverage reported by respondent	Categorical: e.g., Job, Medicaid, Uninsured	ADPH PRAMS Phase 8; 2016–2021; Qn# 9, #10, #11
Household Income	Total reported household income	12 bracketed categories	ADPH PRAMS Phase 8; 2016–2021; Qn# 79

Note: All frequencies reported are unweighted and derived from aggregated ADPH PRAMS Phase 8 data (2016–2021) prior to pseudo-individual expansion using the uncount() function in R 4.4.2.

Primary Variables

*Pregnancy Intentionality*: This was categorized as planned or unplanned based on responses to PRAMS Phase 8 Question 12 (Qn12).

*Early Prenatal Care Initiation*: This was evaluated dichotomously as receiving prenatal care as early as desired (Yes/No), based on PRAMS Phase 8 Question 20 (Qn20).

Barrier Variables in the HCAB Domains

The barrier items from PRAMS Phase 8 Question 21 (Qn21) were categorized within three domains of HCAB:

*Structural Barriers*: This captures barriers relating to transportation, appointment availability, and clinic operational hours.

*Financial Barriers*: This captures barriers relating to insurance status, cost, and affordability issues.

*Cognitive Barriers*: This captures barriers relating to pregnancy recognition, perceived importance of care, cultural beliefs, and health literacy.

Demographic Covariates

Demographic covariates included maternal age, body mass index (BMI), insurance coverage (Qn10, Qn11), race/ethnicity, and annual household income (Qn79), with descriptive statistics presented in the Section 3 (see Table 2).

### 2.4. Statistical Analysis

The following statistical procedures were employed to examine the study’s hypotheses regarding pregnancy intentionality, latent barrier profiles, and early prenatal care initiation. Latent class analysis (LCA) was conducted using PRAMS Phase 8 barrier items to identify distinct barrier profiles among respondents empirically. Model selection was guided by the Akaike Information Criterion (AIC) and Bayesian Information Criterion (BIC), with preference given to models exhibiting lower values and more interpretable class distributions (see Table 3 in the Section 3).

Multivariable logistic regression models evaluated the association between pregnancy intentionality (planned vs. unplanned) and early prenatal care initiation, adjusting for demographic covariates, such as age, BMI, race/ethnicity, and insurance status. To account for potential small-sample bias and assess interaction effects between latent barrier classes and pregnancy intentionality, Firth-penalized multivariable logistic regression was employed (see Table 4 in the Section 3).

Mediation analyses using weighted least squares mean- and variance-adjusted (WLSMV) estimation assessed whether latent barrier profiles mediated the association between pregnancy intentionality and early prenatal care initiation. Direct and indirect pathways were estimated, with significance assessed via Wald statistics (see Table 5 in the Section 3).

*Qualitative Analyses.* Semi-structured interviews were conducted with eight field experts from diverse backgrounds in maternal and child health policy, clinical care, public health, and social services in Alabama. A set of eight open-ended questions was developed in alignment with the three domains of the HCAB model: structural, financial, and cognitive barriers (see Table 6 in the Section 3). These prompts were designed to elicit expert insights into systemic, informational, and logistical factors influencing early prenatal care access.

Each interview was transcribed and analyzed using a structured, deductive coding approach. The transcripts were reviewed line by line, with expert responses coded directly to the corresponding HCAB domains. Considering the descriptive aim of the qualitative phase and its alignment with a pre-established theoretical framework, formal inter-coder reliability testing was not applied. This method facilitated the structured integration of expert perspectives within the context of the quantitative findings.

*Ethical Considerations.* The study protocol was approved by the Institutional Review Board (IRB) at Auburn University under protocol number 24-831, with approval granted on 11 July 2024. Permission to utilize the Alabama PRAMS Phase 8 dataset was obtained from the Alabama Department of Public Health. All expert participants were informed of the study’s purpose and voluntarily agreed to participate in the interviews, which constituted implied consent in accordance with IRB-approved procedures.

## 3. Results

### 3.1. Sample Characteristics

Table 2 details the demographic and clinical characteristics of the study sample (N = 596). A majority of respondents identified as Non-Hispanic White (55.9%), followed by Non-Hispanic Black (33.6%), and those of Hispanic origin (10.6%). The most common BMI classification was healthy weight (42.1%), while a substantial proportion were categorized as obese (33.4%) or overweight (22.5%). Additionally, approximately 42% of participants reported household incomes below 25,000 USD, whereas 29.5% reported annual earnings exceeding 60,000 USD. Readers may refer to Table 2 for a comprehensive distribution of sample characteristics.

### 3.2. Latent Class Analysis

Latent class analysis (LCA) was conducted on the Qn21 barrier items using pseudo-individual-level data simulated from aggregate PRAMS Phase 8 counts. Models specifying two, three, and four latent classes were estimated, with model fit assessed using the Akaike Information Criterion (AIC) and Bayesian Information Criterion (BIC), both of which balance fit quality and parsimony. Lower values indicate improved model performance.

As shown in Table 3, the two-class model yielded the lowest AIC (5940.01) but exhibited a severely imbalanced class distribution, with most cases clustering into a single class. The three-class model had slightly higher AIC (5947.22) and BIC (6100.47) values but provided a more conceptually interpretable structure aligned with the theoretical framework. The four-class solution produced even higher AIC (5955.74) and BIC (6161.52) values, indicating potential overfitting. Based on fit statistics and substantive interpretability, the three-class model was preferred.

To further assess the adequacy of the three-class model, additional selection criteria were examined. The normalized Shannon entropy for the three-class model was 0.217, implying moderate class separation across respondents based on endorsement patterns. Classification accuracy, computed as the mean of maximum posterior probabilities for each respondent, was 0.898, suggesting that 89.8% of respondents were assigned to their most likely class with high certainty. These indicators complement the AIC and BIC values presented in Table 3, supporting the interpretability and reliability of the three-class structure.

**Figure 2:**
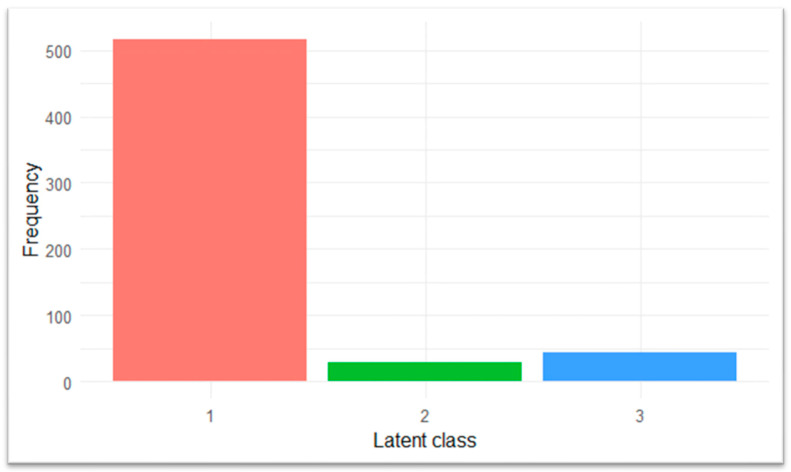
Distribution of latent class membership among respondents based on LCA of Qn21 barrier items. Note: Class 1 (Low Barriers), Class 2 (Predominantly Cognitive Barriers), and Class 3 (Predominantly Structural Barriers) reflect distinct access profiles aligned with HCAB domains. These distinctions highlight which populations may require tailored interventions to reduce informational or logistical barriers to early prenatal care.

**Figure 3:**
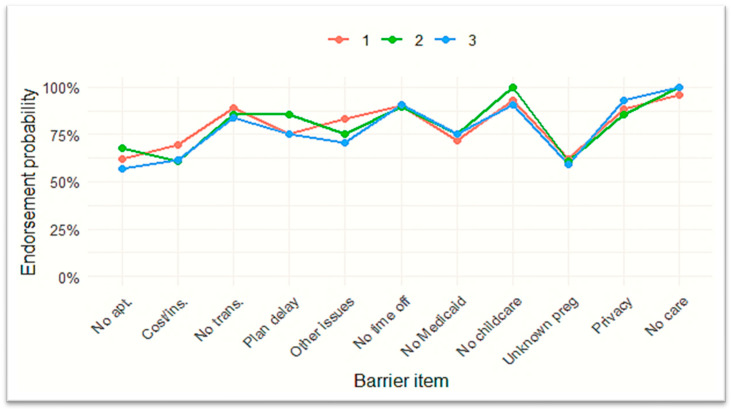
Item-level endorsement probabilities for each latent class derived from Qn21 barrier items. Note: Profiles depict varying combinations of structural, cognitive, and financial barriers. Class 2 highlights personal and informational challenges, while Class 3 emphasizes logistical obstacles. These profiles guide targeted policy responses under the HCAB framework.

This solution identified three distinct latent profiles of access barriers. Class 1 (Low Barriers) represented respondents with minimal endorsement of any barriers. Class 2 (Predominantly Personal Barriers) encompassed individuals who primarily faced cognitive or informational challenges, such as uncertainty about when to seek care or discomfort discussing pregnancy. Class 3 (Predominantly Structural Barriers) displayed high endorsement of logistical obstacles, including transportation difficulties, work conflicts, and cost concerns, aligning with the HCAB framework. Frequencies for latent class membership are presented in Figure 2, and item-level endorsement probabilities for each class are shown in Figure 3.

As shown in Figure 2, the vast majority of respondents were assigned to Class 1 (n = 517 [87.8%]), whereas only a small minority fell into Class 2 (n = 28 [4.8%]) and Class 3 (n = 44 [7.5%]). This distribution shows that most simulated individuals fell into the profile represented by Class 1, whereas only a minority exhibited the barrier patterns captured by Classes 2 and 3. Figure 3 presents the item-level endorsement probabilities that define each latent class profile.

As shown in Figure 3, all three classes exhibited high endorsement for the item, *did not want care*, with values approaching or exceeding 90%. Class 2 (Predominantly Personal Barriers) showed the highest endorsement for barriers related to Medicaid coverage, childcare, and privacy concerns, suggesting difficulty navigating informational and support-related aspects of care. Class 1 (Low Barriers) exhibited moderate endorsement across most items, with cost and scheduling concerns emerging more prominently than cognitive barriers. In contrast, Class 3 (Structural Barriers) displayed elevated endorsement for logistical challenges such as transportation and appointment delays but relatively lower endorsement for insurance-related items. Altogether, the latent classes correspond to distinct domains within the HCAB model: Class 1 (Low Barriers) reflects minimal endorsement of barriers; Class 2 (Predominantly Cognitive Barriers) captures informational and personal challenges, such as uncertainty about when to seek care; and Class 3 (Predominantly Structural Barriers) includes high endorsement of transportation, work-related, and cost-related obstacles.

### 3.3. Firth-Penalized Multivariable Logistic Regression

A Firth-penalized multivariable logistic regression was conducted to examine whether latent barrier profile, pregnancy intentionality (planned vs. unplanned), and their interaction predicted early prenatal care initiation. The model controlled for maternal age, race/ethnicity, and BMI. This approach was chosen to mitigate potential small-sample bias and instability caused by sparse cell sizes in the interaction term. Considering the relatively small number of respondents in latent classes 2 and 3, statistical power to detect mediation or interaction effects was limited, increasing the likelihood of Type II error in these secondary analyses. To provide further interpretive clarity, a post hoc power analysis was performed using the observed effect size from the direct association between pregnancy intentionality and early prenatal care initiation. Based on the fitted logistic regression model, this analysis estimated a post hoc power of 0.99, suggesting a high probability of correctly rejecting the null hypothesis for this direct relationship. This finding supports confidence in the primary association while reinforcing the need for interpretive caution regarding nonsignificant mediation and interaction effects.

As shown in Table 4, neither the main effect of latent class 2 (OR = 0.29, 95% CI [0.00, 2.51], *p* = 0.315) nor unplanned pregnancy (OR = 0.78, 95% CI [0.49, 1.23], *p* = 0.286) significantly predicted early prenatal care initiation. The interaction between latent class 2 and unplanned pregnancy was also not statistically significant (OR = 5.19, 95% CI [0.22, 828.94], *p* = 0.309), underscoring the need to interpret null results cautiously, given the constraints of statistical power.

Additionally, none of the demographic covariates, including maternal age, race/ethnicity, and BMI, were statistically significant. These findings suggest that in this analytic sample, latent class membership, pregnancy intentionality, and their interaction did not significantly influence the likelihood of initiating prenatal care early, though limitations in sample size may contribute to the absence of significant effects.

### 3.4. Mediation Analysis

A path analysis was conducted to evaluate whether latent class membership mediated the association between pregnancy intentionality and early prenatal care initiation. The model was estimated using weighted least squares mean- and variance-adjusted (WLSMV) estimation and included direct, indirect, and total effects.

As shown in Table 5, there was no statistically significant indirect effect of pregnancy intentionality on early prenatal care initiation through latent class membership (indirect effect = 0.012, *p* = 0.518). The direct effect of intentionality on early care also remained nonsignificant (direct effect = 0.067, *p* = 0.221), consistent with prior multivariable logistic regression results. These findings suggest that variation in access barrier profiles does not meaningfully explain the relationship between pregnancy intentionality and early prenatal care initiation. Latent class membership did not serve as a significant mediator in this analytic model.

### 3.5. Qualitative Findings from Expert Interviews

Semi-structured interviews with eight maternal and child health experts provided contextual insights into barriers to early prenatal care access. The interview prompts were developed to align with each domain, as captured in Table 6.

#### 3.5.1. Structural Barriers

Most experts highlighted logistical challenges as persistent structural barriers to timely prenatal care. Frequently cited concerns included inadequate transportation in rural areas, fragmented care coordination, and clinic operating hours that conflict with work schedules. Several experts emphasized that these system-level constraints disproportionately affect women with limited social support or caregiving responsibilities. One expert noted, “*The system is just not designed for the realities of a working mother in a rural county*”.

#### 3.5.2. Cognitive Barriers

Health literacy, awareness, and the perceived importance of care were frequently described as critical influences on care-seeking behavior. Misunderstandings about when to begin prenatal care and fears of judgment or stigma were common among first-time mothers and immigrant populations. Some experts also observed that provider dismissiveness or poor communication erodes trust and discourages follow-up, with one interviewee explaining, “*Women don’t return when they feel unheard*”.

#### 3.5.3. Financial Barriers

Although financial concerns were mentioned less frequently than structural and cognitive barriers, they were still considered significant by several experts. Issues cited included gaps in Medicaid enrollment, delays in coverage activation, and confusion about eligibility. One expert pointed out, “*Even when the care is technically free, women do not know how to access it, or assume they cannot*”.

#### 3.5.4. Cross-Cutting Observations

Experts agreed that while individual motivation, such as pregnancy planning, is vital, often, it is frequently overshadowed by environmental and structural constraints. They suggested that the weak mediation or moderation effects observed in the quantitative phase may reflect the complexity and contextual nature of these barriers. To address these challenges, experts recommended public health interventions such as culturally tailored patient navigation, mobile outreach, and coordinated case management. Others advocated for community-based resource directories and digital health literacy campaigns.

## 4. Discussion

### 4.1. Summary of Key Findings

This explanatory sequential mixed-methods study assessed how pregnancy intentionality and healthcare access barriers influence early prenatal care initiation. Although pregnancy intentionality was associated with higher odds of early care initiation in initial multivariable logistic regression models, this association was not statistically significant after adjusting for covariates or when modeled with interaction terms involving barrier profile membership. The mediation analysis also found no significant indirect effects through latent class membership, suggesting that additional structural and interpersonal factors influence care-seeking behaviors in ways that pregnancy intentionality alone does not fully capture.

However, expert interviews reinforced the importance of structural and cognitive barriers in limiting care access. One expert emphasized that *“The system is just not designed for the realities of a working mother in a rural county”*, highlighting the persistent logistical and scheduling issues women face. Several interviewees emphasized that even when a pregnancy is planned, structural or interpersonal barriers can delay access to care. For instance, one expert noted that the distinction between planned and unplanned pregnancy is insufficient in settings where women are unable to secure timely appointments due to clinic overload or limited availability. This perspective supports the interpretation that scheduling constraints can hinder access irrespective of intentionality.

Another noted that *“Women don’t return when they feel unheard”*, pointing to how poor provider communication and perceived stigma reduce follow-up and trust. Similarly, several participants pointed to distrust of healthcare providers as a barrier that may discourage women from initiating prenatal care, even in wanted pregnancies. One maternal health stakeholder remarked that previous negative encounters, such as experiences perceived as dismissive or biased, can lead to hesitation in seeking early care. These accounts align with the HCAB framework and underscore how emotional and relational dimensions of access influence care-seeking behaviors. These perspectives also align with prior findings that transportation barriers [23], low health literacy [24], and mistrust in providers are major contributors to delayed prenatal care, especially among underserved groups [25,26].

The latent class analysis (LCA) findings revealed three distinct barrier profiles among Alabama women: Low Barriers, Predominantly Personal Barriers, and Structural Barriers. These profiles align conceptually with the HCAB model. Stigma also emerged as a notable theme. One interviewee explained that young or unmarried women, even when intentional in their pregnancies, may avoid early care to escape judgment within clinical environments. These nuances provide insight into why pregnancy intentionality alone may not account for the statistically nonsignificant moderation and mediation results observed in the quantitative data. Additionally, sociocultural factors, interpersonal dynamics, and perceptions of stigma may influence care-seeking behaviors in complex ways that are not easily captured through quantitative indicators.

Altogether, the combined quantitative and qualitative findings emphasize that while pregnancy intentionality may influence care-seeking decisions, entrenched structural, financial, and informational barriers often override care-seeking intent, especially among marginalized populations and other vulnerable populations.

### 4.2. Understanding Barriers Beyond the Numbers

The absence of statistically significant relationships in the adjusted models does not diminish the importance of this study’s findings. Rather, it highlights the complexities of prenatal care access, showing that individual-level variables, such as pregnancy intentionality, do not fully capture the lived experiences of pregnant women navigating healthcare systems in resource-limited contexts. Through the HCAB framework, expert interviews provide key insights into why the intent to seek early care does not always result in timely action. For instance, structural barriers, including transportation limitations [23], rigid clinic hours, and fragmented scheduling systems, remain persistent obstacles to early prenatal care initiation. These logistical challenges are very pronounced in rural areas [5], where inadequate healthcare infrastructure often prevents pregnant women from accessing timely care.

Similarly, cognitive barriers, such as low health literacy [24], uncertainty about when prenatal care should begin, and perceived stigma, further discourage care-seeking behaviors. These challenges disproportionately affect first-time mothers and immigrant women, who may encounter linguistic and cultural barriers or negative past healthcare experiences. Provider communication also plays a significant role, as women who feel dismissed or unheard by medical professionals may delay or altogether avoid follow-up care. Likewise, financial constraints continue to impede access to routine and risk-appropriate prenatal care. For instance, experts noted that delays in Medicaid coverage activation and confusion around eligibility contribute to structural exclusion from essential health services.

A notable implication of these findings is that structural and cognitive constraints may neutralize the effects of personal agency, such as pregnancy intentionality, on early prenatal care-seeking behavior. While readiness and motivation are vital, systemic obstacles can significantly hinder access. Expert interviews reinforce this interpretation, highlighting instances where women eager to begin prenatal care struggle to secure timely appointments due to clinic limitations. Additionally, concerns surrounding stigma and distrust in healthcare providers were evident among younger women and those without insurance, reinforcing how institutional and interpersonal challenges may override individual intention in resource-limited environments.

Although the LCA-derived barrier profiles were not significant mediators or moderators, their alignment with expert themes strengthens their practical relevance. Experts emphasized that these barriers rarely occur in isolation; rather, women often experience overlapping structural, cognitive, and financial challenges that collectively diminish the impact of pregnancy planning. This broader conceptual understanding frames the findings in a way that anchors how systemic inequities constrain individual decision-making, supporting the need for policy solutions that address upstream determinants of care.

Altogether, the HCAB framework provides a compelling structure for understanding how multi-level barriers interact to affect prenatal care access for pregnant women and their families. The findings of this study emphasize that policy-level interventions are essential for converting intentions into timely care for those most affected by systemic disadvantages. Key actions include improving clinic accessibility, enhancing cultural responsiveness, and promoting better communication between providers and patients. Addressing these challenges goes beyond individual behavior change; it is a call to action that requires structural reforms to reduce logistical, informational, and financial barriers to accessing prenatal care.

### 4.3. Implications for Policy and Practice

The findings of this study highlight the urgent need for policy solutions that extend beyond individual behavior change and address the multi-level barriers pregnant women experience in accessing early prenatal care. These policy recommendations draw on empirical patterns identified in the latent class analysis, especially the structural and cognitive profiles. Expert insights provide context for the persistence of these barriers, even in cases where pregnancy was intentional. In particular, structural barriers require robust investments in rural transportation services, expanded clinic hours, and the integration of telehealth for prenatal consultations. At both the state and local levels in Alabama, fostering partnerships with community-based organizations can facilitate patient navigation and case management systems that support women in overcoming logistical obstacles before, during, and after birth.

In addition, improving cognitive and informational access during the perinatal period requires tailored, culturally competent outreach strategies. Health departments and provider networks should embed health literacy interventions into prenatal programs, especially those targeting immigrant populations and first-time mothers. Provider training in trauma-informed, respectful communication is essential, as it can enhance trust and reduce perceived stigma that deters care-seeking.

Addressing the financial barriers requires simplifying Medicaid enrollment processes and removing bureaucratic delays, such as verification procedures and eligibility reviews. When done efficiently, these steps can prevent obstacles or eliminate avoidable gaps in coverage. Policymakers and health analysts should consider continuous eligibility measures for the prenatal and postpartum periods while providing clear guidance to caseworkers and patients. More generally, inter-agency collaboration among healthcare providers, social services, and public health systems is crucial for designing responsive interventions.

In health jurisdictions with regionalized perinatal systems, the evaluation and monitoring of referral and specialty coordination processes remain critical to ensuring timely transitions across levels of care. Existing best practices, such as the medical home model, may serve as a blueprint for integrating primary and specialty services, provided these systems are well-resourced and equitably distributed across diverse care settings. When aligned with HCAB-informed insights, such interventions can help reduce maternal health disparities and improve birth outcomes across varied populations.

### 4.4. Study Limitations

This study has some limitations that warrant discussion. First, the study’s analytic sample was constrained to 596 simulated observations due to the application of listwise deletion for records with missing responses on key variables. While this approach ensured complete data across core predictors and outcomes, it may have resulted in a reduction in sample size and diversity, potentially affecting statistical power to detect smaller effects. While the Firth-penalized multivariable logistic regression method was employed to reduce small-sample bias, some interaction effect estimates exhibited wide confidence intervals, indicating sparse data challenges. This method also addressed concerns about sparse categories, such as the small underweight BMI group, which comprised only 2% of the analytic sample. This does not undermine the validity of the approach but suggests that further research using respondent-linked datasets could provide more precise estimates, especially in analyses requiring mediation or moderation testing.

Second, the absence of sampling weights limits the ability to generalize findings to Alabama’s broader maternal population. While the study offers valuable insights into prenatal care barriers, its conclusions should be interpreted taking into account this methodological constraint. Additionally, pseudo-individual-level data were generated using the uncount() function in R, proportionally expanding aggregate counts into case-level records. While this approach aligns with established LCA methods and provides a reasonable approximation for exploratory modeling, it does not fully replace direct respondent-level microdata. The use of simulated data inherently limits direct comparisons with individual-level respondent datasets, particularly for nuanced subgroup analyses.

Third, latent class analysis (LCA) was conducted on simulated individual-level data derived from aggregated ADPH PRAMS Phase 8 counts. While methodological precedents from past studies support LCA applications using constructed datasets [17,18,19,20], reliance on pseudo-individual records prevents direct linkage to actual maternal respondent data. Consequently, generalizability may be affected, especially in assessing within-group variations that would otherwise be observable in fully respondent-linked datasets.

Despite these limitations, integrating latent class analysis and expert interviews strengthens the study’s contributions by offering a nuanced understanding of access barriers beyond individual-level predictors. Future research should evaluate how structural and cognitive constraints interact with pregnancy intentionality using larger datasets with weighted analyses or alternative imputation methods. Expanding methodological designs to include respondent-linked microdata would further enhance precision in evaluating latent profiles of prenatal care barriers.

## 5. Conclusions

This study examined how pregnancy intentionality and healthcare access barriers influence early prenatal care initiation using an explanatory sequential mixed-methods approach anchored in the HCAB model. Although statistical analyses did not reveal significant mediation or moderation effects, expert interviews provided vital context, showing how structural, cognitive, and financial barriers influence prenatal care decisions in real-world settings.

Considering the entrenched nature of these barriers, addressing disparities in maternal and perinatal outcomes requires coordinated strategies that extend beyond the prenatal period. One promising approach is the expansion of community-based doula programs, which offer emotional, educational, and logistical support to first-time mothers and adolescents. These programs help bridge gaps in health literacy, foster trust in the care process, and facilitate continuity of care before, during, and after pregnancy. Additionally, strengthening Alabama’s perinatal regionalization systems and expanding the medical home model could improve coordination across care settings. More crucially, enhancing early identification of high-risk individuals and ensuring appropriate referrals would support the long-term management of chronic conditions and preexisting health issues.

Further, equitable maternal health outcomes depend on provider-level strategies that prioritize respectful and culturally competent care. Training in trauma-informed approaches, implicit bias awareness, and effective communication can improve patient-provider interactions, especially for historically underserved populations. Strengthening provider accountability in care coordination could encourage timely prenatal engagement with pregnant women. Beyond pregnancy, greater emphasis must be placed on insurance continuity, preconception health, and postpartum care. Closing gaps in coverage and care coordination throughout the reproductive continuum is essential for addressing disparities, not just during pregnancy but well before conception and beyond delivery.

Finally, the integrated findings from the explanatory sequential mixed-methods design offer valuable insights into the barriers that influence care-seeking behavior. For instance, the qualitative component provides further context by showing how structural, cognitive, and interpersonal factors influence real-world experiences. Informed by HCAB principles and enriched by clinical and community expertise, the study findings pinpoint the need for structural, interpersonal, and policy-level approaches that support equitable access to prenatal care.

## Figures and Tables

**Table 2:** Descriptive summary of study variables.

Variable	Category	n (%)
Pregnancy Intentionality	Planned	277 (46.5%)
	Unplanned	319 (53.5%)
Early Prenatal Care	Yes	403 (67.6%)
	No	193 (32.4%)
Race/Ethnicity	Non-Hispanic White	333 (55.9%)
	Non-Hispanic Black	200 (33.5%)
	Hispanic Origin	63 (10.6%)
Body Mass Index (BMI)	Healthy Weight	251 (42.1%)
	Obesity Weight	199 (33.4%)
	Overweight Weight	134 (22.5%)
	Underweight Weight	12 (2.0%)
Annual Household Income	Low (<25 K USD)	249 (41.8%)
	Middle (25 K–60 K USD)	171 (28.7%)
	High (60 K+)	176 (29.5%)
Maternal Age	15–19	38 (6.4%)
	20–24	153 (25.7%)
	25–29	183 (30.7%)
	30–34	147 (24.7%)
	35 or older	75 (12.6%)

Note: Frequencies and percentages are based on the final simulated dataset (N = 596). Intentionality was simulated using Qn13 proportional distributions. Maternal age was updated using reconciled values from Age_Group_collapsed.

**Table 3:** Model fit statistics for latent class solutions (2–4 classes) using Qn21 barrier items.

Number of Classes	AIC	BIC
2	5940.01	6040.71
3	5947.22	6100.47
4	5955.74	6161.52

Note: Lower AIC and BIC values indicate improved model fit. The three-class solution was selected based on conceptual interpretability despite a marginally higher AIC.

**Table 4:** Firth-penalized logistic regression predicting early prenatal care initiation with latent class × pregnancy intentionality interaction.

Predictor	Odds Ratio	SE	*z*-Value	*p*	95% CI
Main effects					
Latent Class 2 (vs. Class 1)	0.287	1.453	−0.86	0.315	[0.002, 2.514]
Pregnancy Intent Unplanned (vs. planned)	0.779	0.231	−1.08	0.286	[0.490, 1.232]
Covariates					
Age 20–24	0.733	0.390	−0.80	0.429	[0.333, 1.585]
Age 25–29	0.674	0.389	−1.01	0.313	[0.307, 1.452]
Age 30–34	1.289	0.366	0.69	0.492	[0.624, 2.679]
Age 35–39	1.152	0.365	0.39	0.701	[0.557, 2.395]
Age 40 or older	0.552	0.425	−1.40	0.161	[0.229, 1.262]
Race Non-Hispanic Black	0.707	0.285	−1.22	0.229	[0.396, 1.243]
Race Non-Hispanic White	0.981	0.270	−0.07	0.943	[0.571, 1.681]
BMI Obesity Weight	0.986	0.323	−0.04	0.965	[0.516, 1.875]
BMI Overweight Weight	0.897	0.316	−0.35	0.732	[0.476, 1.681]
BMI Underweight Weight	0.879	0.315	−0.41	0.687	[0.467, 1.649]
Interaction					
latent class 2 × Pregnancy Intent Unplanned	5.185	1.714	0.96	0.309	[0.222, 828.944]

Note: N = 589. Firth’s penalized-likelihood multivariable logistic regression was employed to reduce small-sample bias. Odds ratios (ORs) and 95% profile-penalized confidence intervals (CIs) reflect the change in odds of initiating prenatal care as early as desired for a one-unit increase in the predictor (or, for categorical predictors, relative to the reference category). Reference categories were latent class 1; planned pregnancy; age < 20 years; Hispanic/other race; healthy-weight BMI. All *p* values > 0.05.

**Table 5:** Mediation of unplanned pregnancy on early prenatal care via barrier–class membership.

Path	B	SE	β	z	*p*
a_1_. Personal barriers ← Unplanned pregnancy	0.035	0.121	0.018	0.29	0.769
a_2_. Structural barriers ← Unplanned pregnancy	0.010	0.214	0.005	0.05	0.962
b_1_. Early care ← Personal barriers	–0.199	0.086	–0.199	–2.31	0.021 *
b_2_. Early care ← Structural barriers	0.242	0.142	0.241	1.70	0.089 †
c′. Early care ← Unplanned pregnancy	–0.111	0.136	–0.055	–0.81	0.416
Indirect via personal barriers (a_1_ × b_1_)	–0.007	0.024	–0.004	–0.29	0.771
Indirect via structural barriers (a_2_ × b_2_)	0.002	0.052	0.001	0.05	0.962
Total indirect (sum of both)	–0.005	0.064	–0.002	–0.07	0.943
Total effect (c′ + total indirect)	–0.115	0.126	–0.058	–0.91	0.361

* *p* < 0.05; † *p* < 0.10. Note: N = 589. Unstandardized coefficients (B) and standard errors (SE) come from WLSMV estimation in lavaan; β are standardized estimates; z is the Wald statistic; p is two-tailed. Indirect effects are defined as a_i_ × b_i_.

**Table 6:** Expert interview questions by HCAB domain.

Interview Question	HCAB Domain(s)
1. What do you see as the biggest structural barriers to early prenatal care in Alabama?	Structural
2. In your experience, how do financial constraints affect women’s access to timely prenatal care?	Financial
3. How do knowledge, awareness, or cultural beliefs shape decisions around seeking early prenatal care?	Cognitive
4. What are your observations on provider–patient communication, particularly in early pregnancy?	Cognitive
5. How does social support, or the lack of it, affect care-seeking behavior?	Structural
6. From your experience, how do limitations in care coordination, clinic infrastructure, or provider availability affect early prenatal care access?	Structural
7. What kinds of interventions or tools would help pregnant women navigate the system efficiently?	Cognitive, Structural
8. Are there any groups of women, like immigrants, low-income, and rural residents, who face unique access barriers?	Structural, Financial, Cognitive

Note: Interview prompts were aligned with the structural, financial, and cognitive domains of the HCAB model to elicit expert insight on prenatal care access challenges.

## Data Availability

Due to the existing data use agreement with the Alabama Department of Public Health, the complete dataset cannot be shared publicly. However, data supporting the study’s findings can be made available upon reasonable request.

## Abbreviations

The following abbreviations are used in this manuscript:

AIC	Akaike Information Criterion
ADPH	Alabama Department of Public Health
BIC	Bayesian Information Criterion
BMI	Body Mass Index
HCAB	Health Care Access Barriers
IRB	Institutional Review Board
LCA	Latent Class Analysis
MMR	Maternal Mortality Rate
PRAMS	Pregnancy Risk Assessment Monitoring System
SE	Standard Error
WLSMV	Weighted Least Squares Mean- and Variance-Adjusted
